# Somatostatin analogues in the treatment of gastroenteropancreatic neuroendocrine tumours, current aspects and new perspectives

**DOI:** 10.1186/1756-9966-29-19

**Published:** 2010-03-02

**Authors:** Marialuisa Appetecchia, Roberto Baldelli

**Affiliations:** 1 Endocrinology Unit, Regina Elena National Cancer Institute, Via Elio Chianesi, 53, Rome 00144, Italy

## Abstract

Gastroenteropancreatic neuroendocrine tumours (GEP NETs) are rare tumours that present many clinical features.

They secrete peptides and neuroamines that cause distinct clinical syndromes, including carcinoid syndrome. However, many are clinically silent until late presentation with mass effects.

In 2000 the WHO developed a new classification which gives a better description of the characteristics and biological behaviour of the tumour.

Surgical resection is the treatment of first choice for a patient with a GEP NET. In metastatic disease multiple therapeutic approaches are possible. In these cases the goal is to improve quality of life and to extent survival.

GEP NETs express somatostatin receptors (SSTRs), which are bound by somatostatin (SST) or its synthetic analogues, although the subtypes and number of SSTRs expressed is very variable.

Somatostatin analogues are used frequently to control hormone-related symptoms while their anti-neoplastic activity, even if it has not been widely studied and the regarding data are discordant, seems to result prevalently in tumour stabilisation.

A few patients who fail to respond or cease to respond to standard SST analogues treatment seem to have a response to higher doses of these drugs.

The use of higher doses of somatostatin analogues or the development of new subtype selective agonists and chimaeric somatostatin analogues, or pan-somatostatin will probably improve the clinical management of these patients.

This review provides an update on the use of somatostatin analogues in the management of GEP NETs and discusses novel clinical strategies based on SSTR 2 gene transfer therapy.

## Introduction

Gastroenteropancreatic neuroendocrine tumours (GEP NETs) are an heterogeneous group of relatively rare tumours, whose yearly incidence is 1.2-3.0 cases/100,000 inhabitants [1].

The database of the National Cancer Institute, Surveillance Epidemiology and End Results (SEER), mirroring the attention standards for US average patients, shows that the age-related incidence of small intestine and digestive tract carcinoids increased by 460% and 720% respectively, within a period of 30 years [2]. GEP NETs arise from local gastrointestinal stem totipotent cells, rather than from the neural crest, as assumed at first [3]. According to the most recent histological classification of tumour features - based on diameter and presence/absence of local or distance metastases - developed by the World Health Organization (WHO), tumours are classified as:

a) well-differentiated neuroendocrine tumours; b) well-differentiated neuroendocrine carcinomas;

c) poorly-differentiated neuroendocrine carcinomas; d) mixed exocrine-endocrine carcinomas [4,5].

In most cases, they are single isolated forms, but they can be multiple and part of familiar syndromes such as MEN 1 syndrome, von Hippel-Lindau disease and neurofibromatosis, type 1. These are mostly (well-differentiated) tumours with relatively slow growth, even if some of them can have an aggressive behaviour (poorly-differentiated carcinomas).

The clinical picture depends on the site of the primary tumour and its ability to secrete neuroamines and peptides at supra-physiological levels (functioning tumours), able to cause a symptomatic response (clinical syndromes). Among functioning tumours, major clinical entities are: carcinoid syndrome, hypoglycaemic syndrome, Zollinger-Ellison syndrome, WDHA (Water Diarrhea-Hypo-kaliemia-Achlorydria) syndrome, glucagonoma syndrome.

However, 90% of GEP NETs do not produce biologically active hormones (non functioning tumours) and therefore the diagnosis is often made too late, in presence of symptoms due to the mass effect and/or the presence of metastases, mainly hepatic metastases [1]. In cases at advanced stages, with a diagnostic confirmation of metastasis, as well as in case of disease progression, the prognosis gets worse. In patients with localised well differentiated neuroendocrine carcinomas, 5-year survival is 60-100%. With regional disease or distant metastases 5-year survival is 40% and 29%, respectively [6].

As a matter of fact, the median survival in these cases is approximately 1 or 2 years.

Around 80% of GEP NETs express somatostatin receptors (SSTRs), located on the cell membrane. There are five different G-protein coupled receptor subtypes (SSTRs 1-5) that are differently expressed in the various types of tumour (Table 1 and 2). Tumours expressing SSTRs often contain one or more receptor subtypes. In addition, recent studies have shown that such receptors are preferably expressed in well-differentiated forms, that some advanced tumours loose particular receptor subtypes while keeping others [7,8], that SSTR subtypes can form homo/heterodimers at the membrane level, to develop new receptors with different functional features [9], and that this receptor "association" may be induced by addition of either dopamine or somatostatin.

**Table 1:** Somatostatin receptors^a ^in neuroendocrine gastroenteropancreatic tumours [%]

	SSTR1	SSTR2	SSTR3	SSTR4	SSTR5
All	68	86	46	93	57
Insulinoma	33	100^b^	33	100	67
Gastrinoma	33	50	17	83	50
Glucagonoma	67	100	67	67	67
VIPoma	100	100	100	100	100
N-F	80	100	40	100	60

VIP, vasoactive intestinal polypeptide; N-F, Non functioning; ^a^Using receptor subtype antibodies; ^b^Malignant insulinoma

[Modified from Oberg K, Annals of Oncology, 2004]

**Table 2:** Somatostatin receptor subtypes mRNA in neuroendocrine tumours.

Tumor	SSTR1	SSTR2	SSTR3	SSTR4	SSTR5
Gastrinoma	79%^a^	93%	36%	61%	93%
Insulinoma	76%	81%	38%	58%	57%
N-F	58%	88%	42%	48%	50%
Carcinoid (gut)	76%	80%	43%	68%	77%

SST, somatostatin receptor; N-F, Non functioning;^a ^Indicates the percentage of positive tumours for each sst. mRNA expression may overestimate the number of receptors present, depending on the technique used [PR-polymerase chain reaction, Northern blot, in-situ hybridization].

[Data from Plöckinger U. Biotherapy. Best Practice & Research Clinical Endocrinology & Metabolism 2007; Vol. 21, No. 1, pp. 145-162]

In a study examining 81 functioning and non-functioning GEP NETs the large parte of the tumours expressed SSTRs 1, 2, 3 and 5, while SSTR 4 was detected only in a small minority [10].

Somatostatin receptors have been extensively mapped in different pancreatic tumours by means of autoradiography, reverse-transcription polymerase chain reaction, in situ hybridization and immunohistochemistry; SSTRs 1, 2, 3 and 5 are usually expressed in pancreatic NETS. Pancreatic insulinomas had heterogeneous SSTRs expression while 100% of somatostatinomas expressed SSTR 5 and 100% gastrinomas and glucagonomas expressed SSTR 2 [11].

Somatostatin (SST) is a natural peptide hormone secreted in various parts of the human body, including the digestive tract, able to inhibit the release of numerous endocrine hormones, including insulin, glucagon, and gastrin. The biological effects of somatostatin are mediated through its specific receptors (SSTR 1-5) with a high degree of sequence similarity (39-57%) and which have been cloned in the early 1990s. They all bind natural peptides, somatostatin 14, somatostatin 28 and cortistatin with similar high affinity (nM range). However, endogenous somatostatin short half-life in circulation (1-3 min), makes it difficult to use it continuously and has resulted in the development of synthetic analogues. By the early 1980s a number of short synthetic analogues of somatostatin including SMS201-995 (octreotide), RC-160 (vapreotide), BIM 23014 (lanreotide), and MK 678 (Seglitide) were developed.

These cyclic octapeptides are more resistant to peptidases and their half-lives and hence their biological activities are substantially longer than native somatostatin (1.5-2 h vs 1-2 min) [12].

The development of a depot formulation of octreotide, Sandostatin LAR (Novartis) (long-acting repeatable), administered up to 30-60 mg once every 4 weeks has to a large extent eliminated the need for daily injections. Lanreotide (Somatuline; Ipsen, Slough, UK), a long-acting somatostatin analogue administered every 10-14 days, has a similar efficacy to octreotide in the treatment of carcinoid tumors, but its formulation is easier and more comfortable for patients to use [13]. A new slow-release depot preparation of lanreotide, Lanreotide Autogel (Ipsen), is administered subcutaneously up to 120 mg once a month [14].

Native SST and its synthetic analogues show different affinity for the five specific receptor subtypes [9,10,15]. Native SST binds all the five receptor subtypes (SSTRs 1-5). The effects of the SST analogues are mediated by the interaction with SSTR 2 and 5 receptors while the new somatostatin analogue, pasireotide (SOM 230), shows higher binding capacity towards SSTRs 1, 2, 3 and 5 with no agonist activity at the type 4 receptor [15] (Table 3). The different receptor subtypes binding affinities seem to result in different biological and clinical activities. Octreotide is, for instance, 45 times more potent in inhibiting growth hormone (GH) secretion and 11 times more potent in inhibiting glucagon secretion than native SST [10].

**Table 3:** Somatostatin receptor subtype-binding affinity of somatostatin analogues.

Receptor subtype affinity [IC50, nM]					
**Compound**	**SSTR1**	**SSTR2**	**SSTR3**	**SSTR4**	**SSTR5**

SMS-14	2.26	0.23	1.43	1.77	0.88
SMS-28	1.85	0.31	1.3	ND	0.4
Octreotide	1140	0.56	34	7030	7
Lanreotide	2330	0.75	107	2100	5.2
Pasireotide	9.3	1	1.5	>100	0.16

SMS, Somatostatin; ND, not determined.

[Data from Grozinsky-Glasberg S., Endocrine-Related Cancer 2008 Sep;15[3]:701-20].

## The symptomatic and biochemical effects of SST analogues

The initial treatment of GEP NETs is, where possible, always an aggressive surgical approach, aimed at obtaining a curative tumour ablation, even in the presence of metastatic disease. However, in patients with functioning or metastatic tumours, the treatment goal is to improve their quality of life, while monitoring or alleviating the tumour-associated symptoms and increasing survival.

Recently, the diagnostic and therapeutic approach of GEP NETs has considerably improved, mainly due to better imaging techniques (CT, MRI, PET) and somatostatin analogue-based imaging methods, as well as receptor subtype characterisation and the introduction of long-acting somatostatin analogues.

Somatostatin receptor scintigraphy (SRS, OctreoScan^®^), (e.g. ^111^In-pentetreotide) can visualise *in vivo *tumours and metastases that express the somatostatin receptor subtypes 2, 3 or 5 [16] except for metastatic insulinomas, of which only 50% express SSTR 2. Imaging by SRS is not dependent on endocrine function of a NET but is determined by the tumour's endowment of SSTRs. This somatostatin analogue-based imaging method may help to decide which patients are suitable for treatment with somatostatin analogues (octreotide or lanreotide), or for tumour-targeted radioactive therapy with radiolabelled somatostatin analogues [13,17-22]. Its overall is high, ranging from 86% to 95% for gut carcinoid tumours to 75-100% for pancreatic endocrine tumours [21,22]. The uptake of radiolabeled octreotide is also predictive of clinical response to therapy with somatostatin analogues.

Since 1980, SST analogues have been used to symptomatically control GEP NETs, especially carcinoids and VIPomas [11,13]. Usually, the treatment with long acting preparations of SST analogues consists in an intramuscular injection (i.m.) every 2 or 4 weeks (octreotide long-lasting, 10-30 mg, LAR; lanreotide long-lasting 60-120 mg LA). In the course of the first two months, additional subcutaneous (s.c.) administrations of short half-life octreotide may be required before achieving such properly stable blood levels of the long half-life synthetic analogue, as to allow adequate symptom control. Their efficacy in the control of symptoms is well-documented [2,12,13], even if patients with islet cell tumour often show a transient (median time 2.5 months) and non-significant response. These are safe and well-tolerated drugs, in both long- and short-term treatments [23-27]. However, after 9-12 months, drug resistance often spreads and patients may show symptom recrudescence. In such cases, the approach proposed was to continue the treatment, by increasing the analogue dosage (for octreotide with gradual increments of 10 mg every 28 days up to 60 mg every 28 days) or, by shortening the administration range by a week [28], if the symptomatologic escape occurs in the week before the next drug injection.A randomised double-blind trial compared long- acting octreotide LAR at 10, 20, and 30 mg every 4 weeks with open-label short-acting octreotide every 8 h for the treatment of carcinoid syndrome. It showed that the efficacy of short-acting octreotide and of the long-acting octreotide-LAR was the same once circulating octreotide steady-state concentrations were achieved [29].

O'Toole *et al *in a multicentre study on 33 patients with the carcinoid syndrome comparing the treatment with lanreotide (30 mg i.m. every 10 days) versus octreotide (200 μg s.c. twice or thrice daily) founded no significant differences in controlling symptoms; 53.8% and 45.4%, respectively, of the patients treated with lanreotide referred disappearance or improvement in flushes and diarrhoea, while these symptoms were observed in 68% and 50%, respectively, of patients on octreotide. Lanreotide and octreotide may also significantly lower the levels of urinary 5-hydroxyindoleacetic acid (5-HIAA), the catabolite of serotonin [30].

Ruszniewski *et al *evaluated the efficacy and safety of the 28-day aqueous prolonged release formulation of lanreotide in 75 patients in a 6-month dose-titration study. Thirty percent of patients showed a biochemical response and 75% and 80% of patients reported resolution of diarrhea and flushing, respectively, which is comparable with the reported effects of other lanreotide preparations. The median decrease in levels of urinary 5-HIAA and serum chromogranin A was 24% and 38%, respectively [31].

An interim analysis of a phase II trial of SOM230 in 21 patients with metastatic carcinoid tumours whose symptoms (diarrhea and flushing) were refractory/resistant to octreotide LAR showed symptom relief in 33% [32].

Approximately 10-15% of patients with midgut carcinoids suffer from watery diarrhoea, flushing, right-sided heart failure and bronchial constriction (carcinoid syndrome), due to the tumour hypersecretion of a variety of endocrine substances, the most frequent of which are serotonin (5-hydroxytryptamine) and the tachykinins [33,34], and therefore somatostatin analogues are important palliative tools for these patients. In insulinoma it has been noted that octreotide treatment may make hypoglycemia worse in those patients lacking SSTRs 2 and 5, and, as glucagon secretion is also inhibited, patients have to be observed closely at the beginning of therapy to prevent severe hypoglycemia due to the reduced glucagon-dependent counter-regulation [35]. Hence, this treatment has to be started in a hospital setting, and should be reserved for only the minority of insulinoma patients with positive imaging on SRS.

Vezzosi *et al *recently assessed that octreotide was effective in the control of hypoglycaemia in more than 50% of the insulinoma patients. The treatment was effective in all SSTR 2 positive patients and in a few SSTR 2 negative ones, while no relation between treatment effectiveness and the expression of SSTR 5 was observed [36]. These results are in concordance with other case reports and smaller series of insulinoma patients reported in the literature [37-41].

In glucagonoma patients somatostatin analogue treatment is indicated for alleviating the symptoms related to the characteristic skin rash (necrolytic migratory erythema) or diarrhoea [42-46].

In somatostatinomas symptoms are due to somatostatin hypersecretion (hyperglycaemia, cholelithiasis, diarrhoea and steatorrhoea, hypochlorhydria) or to the mass effect [47]. Although it seems a paradox to treat patients with symptoms related to elevated SST levels with a somatostatinoma, in 1998 Angeletti et al showed that octreotide treatment was effective in reducing plasma levels of somatostatin and improving the related symptoms in three patients with metastatic somatostatinomas [48].

Recently, have been described nine cases of VIP-oma in which octreotide was very successful as adjuvant therapy for symptoms control and for reducing the serum elevated VIP levels improving the diarrhoea and the electrolyte imbalance [49-51].

## The anti tumour effects of SST analogues

The antineoplastic activity of somatostatin analogues has been demonstrated in several experimental models *in vivo *and *in vitro *[52-57]. However, there is still little known regarding the antiproliferative role of SSA in GEP NETs, although increasing data suggest that such analogues can be tumouristatic, at least in some circumstances [58].

The antineoplastic action of SST analogues depends on the kind of tumour and the receptor subtypes they are bound to, and occurs through direct and indirect mechanisms. While direct activities are mediated by specific membrane receptors and include antimytotic and apoptotic effects, indirect effects do not depend on the receptor bonging and encompass the growth factor inhibition, antiangiogenic and immuno-modulating activities. As a matter of fact, SST analogues are able to inhibit the growth of Swarn chondrosarcoma, used as experimental model of SSTR free tumour [59].

The mitosis inhibition is mediated by SSTRs 2 and 5 and results in the cell cycle arrest [55]. The loss of the SSTR 2 expression in some human adenocarcinomas seems to be responsible for loosing the regulation of cell proliferation [8]. The loss of SSTR 2 may consequently promote tumour growth and make it clear the therapeutic inefficacy of SST analogues in such kind of neoplasia. Apoptosis [programmed cell death] seems to be induced by two different processes: interaction with the SSTR 3 [53] and inhibition of the Insulin-like Growth Factor I (IGF I), potent antiapoptotic hormone [60]. The pro-apoptotic activity of SST analogues seems to have clinical relevance, as shown by the interesting findings published by Eriksson *et al*. that reported an increase in apoptosis in bioptic samples of tissues by patients with GEP NETs, after the treatment with SST analogues at high doses. It followed that apoptosis is related to the biochemical response and the disease stabilisation (70% of cases) [61,62].

However, Faiss *et al*. observed an overall response rate (ORR) of 6.7%, comparable to that recorded at conventional doses [63], in 24 patients with GEP NETs treated with high doses of lanreotide (15 mg/day).

The indirect antiproliferative efficacy of SST analogues is shown by an antiangiogenic mechanism. Angiogenesis, that is the growth of new blood vessels, is essential for tumour growth and metastasis spread. Consequently, the growth can be actually controlled by reducing the vascularisation of the neoplastic tissue. In experimental models, octreotide shows a strong antiangiogenic effect, which is probably mediated by the inhibition of the Vascular Endothelial Growth Factor (VEGF) [64-66]. The response to the treatment with octreotide would result in a significant reduction in VEGF levels compared to the baseline, since it is related to patients' survival [66]. It was observed that standard endothelial cells do not express the SSTR 2 that is present on the contrary, when they proliferate in order to form blood vessels. This could represent further opportunity to treat patients with octreotide that is able to recognise and inhibit new vessel formation both alone and with other drugs, thanks to its high affinity with such receptor (Table 3). Immunomodulation is another indirect mechanism of action of SST analogues. Preliminary evidence suggests that they stimulate the production of immune system components with antitumour effect, such as *natural-killer *cells [67,68], even if up to now it is not clear whether this can be clinically significant thus helping the antitumour efficacy of SST analogues. Few data exists on the functions mediated by the SSTR 4.

However, no unanimity exists about the SST analogue ability to control (i.e. to slow) the tumour progression.

*In vitro *studies reported that the response of different cell lines to the octreotide exposition produces a biphasic dose-response curve [69,70]. Consequently, overdose or underdose of SST analogues may result in a suboptimal antineoplastic activity. Nevertheless, the negative results of some clinical studies in terms of tumour response could be due to the administration of too low doses to achieve receptor optimal saturation. After all, in other studies that used octreotide doses higher than 8 mg/day and lanreotide doses higher than 10 mg/day [71], no improvement of the SST analogue antitumour effect was observed. No study on the tumour response monitored plasma levels of an SST analogue up to now, in order to assess that optimal drug therapeutic levels are reached but not exceeded [72]. Clonflicting results have given with regard to tumour regression. Tumour shrinkage was demonstrated in less than 10% of the patients. However, a stabilisation of tumour growth occurs in up to 50% of the patients with neuroendocrine tumours of various locations. Stable disease was observed in 37-45% of the patients with documented tumour progression before SSA therapy (Table 4). The median duration of stabilisation was 26.5 months [26,73-76]. In a study on a select group of patients with progressive disease, in the 47% of cases was demonstrated a stable disease when treated with a high dose of lanreotide (3-5 g/day) [77]. This result has been confirmed in patients with advanced midgut carcinoids, who had a stabilisation of the disease for 6-24 months in the 75% of cases [78]. One patient with a pancreatic primary tumour, and distant extrahepatic metastases, showed a poor response to treatment in multivariate analysis. Age, size of the primary tumour, and Ki67 did not influence the response rate to SSA therapy [76]. A stabilisation of the disease was maintain throughout long-term follow-up in patients who achieve it after 6 months of treatment; these patients live longer than those unresponsive to therapy [76,79].

**Table 4:** Antiproliferative effect of somatostatin analogues in patients with progressive disease.

SSA	Dosage	N	CR	PR	SD	PD	References
Lanreotide	3000 mg/day	22	0	1	7	14	[97]
Lanreotide	30 mg/2 weeks	35	0	1	20	14	[90]
Octreotide	600 and 1500 mg/day	52	0	0	19	33	[74]
Octreotide	1500 and 3000 mg/day	58	0	2	27	29	[26]
Lanreotide	15000 mg/day	24	1	1	11	11	[97]
Octreotide	600 mg/day	10	0	0	5	5	[73]
Octreotide	median dose of 250 μg three times daily	34	0	1	17	0	[75]
Octreotide LAR 30/ Lanreotide SR	60 mg/28 days	31	0	0	14	4	[76]
							
Total		256	1	6	115	105	
Percentage	(%)		0.3	2	45	41	

SSA, somatostatina analogues; CR, complete remission; PR, partial remission; SD, stable disease; PD, progressive disease.

Very recently Rinke et al performed for the first time a placebo-controlled, double-blind, phase IIIB study in 85 patients with well-differentiated metastatic midgut NETs using octreotide LAR 30 mg intramuscularly in monthly intervals. Median time to tumour progression in the octreotide LAR and placebo groups was 14.3 and 6 months, respectively. After 6 months of treatment, stable disease was observed in 66.7% of patients in the octreotide LAR group and 37.2% of patients in the placebo group. Functionally active and inactive tumours responded similarly. The most favorable effect was observed in patients with low hepatic tumour load and resected primary tumour. Octreotide LAR significantly lengthened time to tumour progression compared with placebo in patients with functionally active and inactive metastatic midgut NETs [80].

Midgut carcinoids express somatostatin receptors in 80 to 100% of cases. SSTR 2 is the most frequently expressed [34]. The antiproliferative effect of somatostatin analogues on the growth of the midgut carcinoids is unknown. A partial or complete responses were observed in less than 10% of the patients, while stabilisation of tumour growth was noticed in 24-57% of the patients [6].

Few data are available regarding the role of somatostatin analogues in the treatment of gastrinomas. In a study of 15 malignant gastrinoma, in about 50% of these patients, octreotide had an antiproliferative effect, including one patient with tumour regression and seven patients with tumour stabilisation [mean period 25 months] patients [81].

The long-acting somatostatin analogue octreotide-LAR was administered in a patient with multiple type A gastric carcinoids for a period of 9 months with a normalisation of serum gastrin levels and permanent disappearance of the tumour [82]. Fykse et al. treated five patients with hypergastrinaemia and gastric carcinoids for a period of 1 year with monthly injections of octreotide-LAR with a significant reduction in tumour load, ECL cell density and normalisation of circulating chromogranin A levels, indicating a possible direct antiproliferative effect of the treatment [83]. These results suggest that the somatostatin analogues could have an important antiproliferative effect. However, data on the effect of somatostatin analogues on tumour growth in patients with gastric carcinoids type C or poorly differentiated endocrine carcinomas are scanty. In poorly differentiated gastric carcinomas, treatment with somatostatin analogs is not considered.

As surgical excision is the definitive treatment of insulinoma, there are few contrasting data in the literature regarding the inhibitory effect of the somatostatin analogues on the growth of these tumours. Grozinsky-Glasberg *et al *have conducted a study regarding the effects of somatostatin analogues on cell proliferation in the rat-derived insulinoma cell line (INS1). Their preliminary data show that octreotide has a significant inhibitory effect on cell proliferation, as assessed by cell counting and MTS assay, and on phosphorylation states of a number of proteins in the PI3K/Akt/mTOR pathway [84,85]. In his work, Vezzosi founded that despite achieving hypoglycaemic control, insulinoma size remained unchanged or increased moderately despite normal blood glucose levels, concluding that somatostatin analogues, as medical treatment is not sufficient to prevent tumour growth in patients with malignant insulinomas [36].

In 2006, Romeo *et al *reported a complete clinical remission with regression of the metastatic lesions in the liver after one year in a patient affected by metastatic insulinoma with severe hypoglycaemia treated with octreotide LAR [85].A more controversial area concerns the treatment of patients with non-functioning endocrine tumours of the pancreas as few studies have been published in these patients. The prospective German Sandostatin multicentre phase II trial investigated the effects of octreotide for one year on tumour growth in 103 patients and included 15 patients with diagnosed non-functional pancreatic tumours [74]. Only 3 out of these 15 patients had a stable disease, in 8 patients a tumour progression occurred while the outcome of the remaining four patients was not clear. As previously said, the SST analogue efficacy depends on the tumour receptor expression patterns, but these are rarely assessed, even if there is evidence of better results on survival obtained with selective treatments. An antiproliferative effect was achieved on hepatic metastatic cells in a patient with a carcinoid tumour, selected for the treatment with SST analogues after the immunohistochemical identification of the SSTR 1, 2 and 5 subtypes expression on the neoplastic cell surface [86]. A complete clinical remission with regression of the metastatic lesions in the liver after one year of treatment was observed in a patient affected by metastatic insulinoma with severe hypoglycaemia treated with octreotide LAR expressing at immunohistochemical analysis of tissue specimens a strong membrane immunoreactivity for SSTR 2 in both the primary nodule and the metastases [85]. However, another study showed neither an antineoplastic effect nor an increase in survival percentage of treated patients [87].

It has been reported that in glucagonoma patients there are no data available on their SSTR expression patterns [45]. In 2006 we demonstrated, for the first time, a scattered immunopositivity for somatostatin receptors in a case of malignant glucagonoma.We had access to polyclonal antibodies specifically targeted against SSTR5 and SSTR2 and we were therefore able to localise these two receptors in our histological sections. The immunopositivity was detected for both receptor subtypes in the membrane and in the cytoplasm of glucagonoma cells. We then treated our patient with a combination therapy consisting of the somatostatin analogue octreotide and interferon-α. The patient had a complete resolution of skin rash, normalisation of plasma glucagon, chromogranin A and neuron specific enolase levels, and metastatic disease stabilisation. The patient's quality of life significantly improved, and she was alive 40 months after debulking surgery [46].

In conclusion, in many cases authors did not stratify patients in treatment arms, according to the histological presence of the SSTR 2 receptor or its clinical expression. Consequently, most of them were likely not to be treated with the optimal drug required to achieve appropriate receptor saturation.

## The effects of higher than usual dose of SST analogues

It was suggested that higher than usual dose of somatostatin analogues treatments (>3,000 μg/day) may promote the anti-proliferative effect, especially in those patients responding to standard doses [2,15,16,78,88,89]. An high-dose treatment with lanreotide (up to 12 mg/day) produced tumour size reduction in 5% and stabilisation in 70% of the 19 patients. In responding patients was observed an induction of apoptosis in the tumours, a phenomenon not seen with regular doses of somatostatin analogs, but often produced by chemotherapeutic agents [62]. Subcutaneously injections of 5 mg lanreotide three times a day for a period of 1 year produced one complete and one partial remission in 30 patients with functional midgut NETs; stable disease in 11 patients (36%) and progression of the disease after 3-12 months of treatment in 11 patients [63]. The treatment with high-dose somatostatin analogues induced apoptosis in neuroendocrine tumours, while this was not found during treatment with low-dose somatostatin, in a study where biopsy specimens were taken before and during somatostatin analogue treatment [61].

In a highly select group of patients with progressive disease, 47% of the patients demonstrated at least stable disease when treated with a high dose of lanreotide (3-5 g/day) [77].

High-dose formula of octreotide has been recently reported to stabilize hormone production and tumour growth in 75% of patients with advanced midgut carcinoid tumours and progressive disease with stabilisation for 6-24 months, [78].

These effects may be attributable to SSTR 2 which is the most frequently expressed subtype and/or SSTR 5, 1 and 3 which are also expressed [90,91].

Data from a study with ultra-high dose octreotide pamoate (Onco-LAR; Novartis) at 160 mg intramuscularly every 2 weeks for 2 months followed by the same dose once monthly, appear to show some promise. Tumour size stabilisation was obtained in 12 patients, a biochemical responses in 9 patients and/or stability in 11. No significant tumour reduction was noted. At 6 months, the median plasma concentrations of octreotide were 25-100 times higher than those obtained by using octreotide LAR at regular doses. A significant inhibition of angiogenesis was also showed through the down-regulation of proliferative factors such as vascular endothelial growth factor (VEGF) and fibroblast growth factor [12]. The highest response rates were reported using octreotide in doses greater than 30 mg/day or lanreotide in doses greater than 5 mg/day (and up to 15 mg/day) [63].

Tomassetti *et al*. have reported that after one-year therapy, the tumour completely disappeared in three patients suffering from gastric carcinoid, two of whom were treated with lanreotide 30 mg i.m. every 10 days [92].

Cirillo in his retrospective study on 165 patients with digestive NETs confirmed that somatostatin analogs can have a role in the treatment of digestive neuroendocrine tumors with low grades of malignancy, a low cellular proliferation index and a high specific receptorial density in vivo, showing a SD ranging from 60 to 66%. Moreover, an increase of the dosage of somatostatin analogs seems to have a better control both of the disease progression and the chronic refractory diarrhea [24].

## Somatostatin analogues and interferon

The combination of SSAs and interferon (IFN) has been used in an effort to enhance the antiproliferative effect of interferon therapy, to add the positive effect of SSAs on hypersecretory syndromes, and to reduce the dose of IFN and thus the number of IFN-related side-effects.

Whether somatostatin analogues and IFN show a synergistic effect on tumour growth and in carcinoid syndrome symptom management is matter of debate. The combination therapy with somatostatin analogues and IFN is in fact limited by the small number of trials, with variable results.

This combination seems of benefit in patients where the usual octreotide treatment failed to achieve a biochemical and symptomatic control [93].

This combination therapy leaded to a significantly lower risk of progressive disease compared with somatostatin analogues alone, and had a higher median survival (51 *vs *35 months) [94]. An anti-proliferative effect of the addition of α-interferon to octreotide was showed in a subgroup of patients with advanced metastatic disease unresponsive to octreotide monotherapy, and prolonged survival was reported in the responder group [95]. However, most published data do not support a major effect of interferons over and above that of somatostatin analogues. In a prospective multicenter study on the effect of combination therapy, Faiss et al showed no advantage on either biochemical or antiproliferative results, while the number of side-effects increased [96].

## Novel somatostatin analogues

Recently the universal or "pan-receptor" somatostatin ligand pasireotide (SOM230) has been developed, which possess high affinity binding to SSTs 2, 3 and 5, moderate affinity for SSTR 1. Its receptor binding profile is 30- to 40-times higher for SSTR 1 and SSTR 5 than octreotide. In a multicentre study on metastatic carcinoid tumours patients whose symptoms (diarrhoea and flushing) were refractory to octreotide-LAR, pasireotide at dosages between 450 μg and 1200 μg twice a day effectively controlled symptoms in 33% of these patients [97]. These results support the hypothesis that pasireotide may have potential in the treatment of these tumours. Subtypes of somatostatin and dopamine receptors may form homo- and hetero-dimers at the membrane level, and this receptor "association" may be induced by addition of either dopamine or somatostatin. Recently, subtype selective analogues and antagonists, as well as bi-specific and hybrid somatostatin/dopamine compounds, binding to SSTR 2, SSTR 5 and dopamine 2 receptors have been developed [98]. Their effects have been studied in several animal and human cell lines, and also in primary cultures from human tumours. Regarding their activity the literature is scanty. Further studies are needed to understand their complex and heterogeneous effects. Chimeric somatostatin-dopamine compounds (dopastatins) with high affinity for SSTRs 2 and D2 receptor (D2R) (BIM-23A387) or to SSTRs 2, 5 and D2R (BIM-23A760) have been showed to inhibit cell proliferation of the non-small-cell lung cancer cell line Calu-6, which expresses SSTRs 2, 5 and D2R with higher potency and efficacy than SSTR 2 and D2R analogues [99]. BIM23A760 can also inhibit ECL cell proliferation with similar potency but with higher efficacy than lanreotide and D2R analogue [9]. The enhanced potency/efficacy of BIM-23A387 and BIM-23A760 may in part be due to the high affinity of these compounds for SSTR 2. However, SSTR 2 can heterodimerize with SSTR 5, and SSTRs 2 and 5 can form heterodimers with D2R which can alter receptor ligand binding affinity and/or signaling and/or receptor trafficking [100-102]. The presence of SSTRs in a higher density in NETs and their ability to form a receptor-ligand complex, can permit the internalisation and the accumulation of radiopharmaceutical inside the tumour [103].

A novel targeted cytotoxic somatostatin octapeptide conjugates such as RC-121 and RC-160 coupled to doxorubicin or its superactive derivative, 2-pyrrolino-DOX (AN-201) was synthesised from Schally and coworkers [56]. AN-238, which contains AN-201 linked to carrier RC-121, has been demonstrated to suppress the growth of Hs746T and NCI-N87 human gastric cancers, which display a high concentration of SSTRs 2 and 5 and seems to target vascular SSTRs in a xenograft tumour model derived from SSTRs negative tumour cells [56].

Another cytotoxic somatostatin analog termed JF-10-81 has been synthesized by Coy and coworkers. This somatostatin analogue, conjugated to camptothecin, inhibits prostate cancer PC-3 cell invasion through a signaling pathway involving PI3K, integrin αVβ3/αVβ5 and matrix metalloproteinases 2 and 9 and exhibited anti-invasive and anti-angiogenic properties *in vivo *[103].

SSTRs are able to form a receptor-ligand complex, that permit the internalisation and the accumulation of the radiopharmaceutical inside the tumour.

Peptide-receptor radionuclide therapy (PRRT) represents an important treatment strategy for tumours that express adequate densities of SSTRs and has proven to be safe and effective.

It was initially performed using indium-111 [19,104]. Recently, the development of somatostatin peptides with higher receptor affinity conjugated with radio-metal labelling chelators, such as DOTA (1,4,7,10-tetrazacyclo-dodecane-*N*, *N'*, *N"*, *N"'*-tetraacetic acid), which may be allow stable labelling with gallium, yttrium or lutetium, changing the affinity profile for particular subtypes of SSTRs can permit new therapeutic options [105]. Waldherr et al evaluated the tumour response to targeted irradiation with the radiolabelled somatostatin analogue ^90^Y-DOTATOC in 41 patients with GEP NET and bronchial tumours. They reported an overall response rate of 24%. For endocrine pancreatic tumours it was 36%. A complete remission was found in 2%, a partial remission (PR) in 22%, a minor response in 12%, stable disease in 49% and progressive disease in 15% of patients. The treatment was well tolerated and there was a significant reduction of symptoms and the 2-year survival time was 76 ± 16% [106].

177Lu DOTATATE [177Lu]DOTA-Tyr(3)-octreotate, a selective analogue of SSTRs 2. In spite of its favourable affinity profile, at its maximum tolerated dose, it is limited by toxic effects on the kidney and bone marrow. Nevertheless, the results seem encouraging compared with historical therapeutic data [107].

Kwekkeboom *et al *obtained promising results using 177Lu DOTATATE [177Lu]DOTA-Tyr(3)-octreotate in 131 patients with NETs. A complete remission was observed in 2% of patients, a partial remission in 26%, a minor response in 19%, stable disease in 35%, and progressive disease in 18% of patients. Higher remission rates were positively correlated with high uptake on pre-therapy SSTRs imaging, whereas progressive disease was significantly more frequent in patients with extensive disease. Median time to progression was more than 36 months [19].

The combination of ^90^Y- and ^177^Lu-labeled analogues [108] seems to have had superior antitumour effects when compared with either ^90^Y- or ^177^Lu-analogue in animals presenting with tumours of various sizes. It has been reported that ^177 ^Lutetium may be more effective for smaller tumours whereas ^90^yttrium may be more effective for larger tumours [109,110].

Recently, the high expression of SSTRs on gastrinomas has been considered as an opportunity to use radiolabeled somatostatin analogues, in order to achieve a cytotoxic effect [^111^In-labelled analogues, ^90^yttrium or ^177^lutetium] [111].

Novel strategies based on SSTRs 2 receptor gene transfer to target tumour growth and angiogenesis represents a new advance in the treatment of unresectable pancreatic tumours.

Buscail et al initially demonstrated that in human pancreatic adenocarcinoma SSTR 2 expression was specifically los[8].

Once gene defect corrected, cell growth as well as tumorigenicity, were significantly reduced in the absence of exogenous ligand [112]. The synthesis and secretion of the natural ligand somatostatin-14 by sst2-transfected cells was responsible for an autocrine/paracrine inhibitory loop [57].

Several study conducted on pancreatic adenocarcinoma animal models demonstrated that intratumoural SSTR 2 gene transfer (using polyethylenimine synthetic vector) inhibited intratumoural production of somatostatin that was critical for the SSTR 2 antitumoral effect.

Primary tumour growth and angiogenesis were highly decreased and associated with a reduction in microvessel density, inhibition of intratumoural production of VEGF and up-regulation of antiangiogenic SSTR 3 receptor expression in peripheral tumour vessels [32,113,114].

## Conclusion

Neuroendocrine tumors of the gastroenteropancreatic (GEP NETs) system comprise a rare group of malignant neoplasms. The somatostatin analogues have been shown to be very useful for symptomatic and biochemical improvement in patients with these tumours while preclinical and clinical studies provide conflicting results on their antitumour effects. The mechanisms of these effects are unknown, but probably are in part due to direct effects on proliferative signalling pathways, activation of apoptosis, and effects on angiogenesis.

Biological response to somatostatin analogs depends on distribution and level of expression of SSTRs subtypes in tumours, and the expression of selective somatostatin receptor-signaling pathway molecules.

The high density of SSTR2 in endocrine tumours explains the use of SSTR 2 specific analogues in the diagnosis and treatment of these tumours. However, the role of SSTR1,3 and 5 appears to be of increasing interest.

The development of new peptidic and non-peptidic somatostatin analogues, subtype selective agonists, chimaeric analogues, or pan-somatostatin analogues will probably improve the diagnosis and treatment of GEP NETs, which express somatostatin receptors other than SSTR 2.

The combination of SSAs and IFN seems of benefit in patients where the treatment with somatostatin analogues alone failed to achieve a biochemical and symptomatic control while their synergistic effect on tumour growth is still unknown.

The analysis of the SSTR status specifically for each patient, and studies of individual tumour biological behaviour, might be of therapeutic interest and could help to optimise treatment expecially in unresectable tumours.

Peptide-receptor-targeted radiotherapy for advanced disease using radiolabeled octapeptide analogues appears to be a significant progress in the treatment of GEP NETs but data are limited, mainly about the best time for its administration, or what is the most appropriate radioligand/combination to be used for each patient, and if and how the doses should be fractionated.

Novel strategies based on SSTR 2 receptor gene transfer to target tumor growth and angiogenesis might offer new prospectives of therapeutic interest mainly to treat unresectable tumours.

Prospective studies including large number of patients regarding the optimal dosage and modes of administration of somatostatin analogues and the development of new slow release, SSTR subtype specific compounds are needed.

## Conflict of interest statement

We disclose any financial and personal relationships with other people or organisations that could inappropriately influence (bias) their work.

## Authors' contributions

MA and RB read and approval the final manuscript.
